# 4-Hy­droxy-1,2,6-tri­methyl­pyridinium bromide monohydrate

**DOI:** 10.1107/S1600536813013330

**Published:** 2013-05-22

**Authors:** T. Seethalakshmi, S. Manivannan, S. Dhanuskodi, Daniel E. Lynch, S. Thamotharan

**Affiliations:** a Department of Physics, Government Arts College (Autonomous), Karur 639 005, India; b Carbon Nanomaterials Laboratory, Department of Physics, National Institute of Technology, Tiruchirappalli 620 015, India; c School of Physics, Bharathidasan University, Tiruchirappalli 620 024, India; d Faculty of Health and Life Sciences, Coventry University, Coventry CV1 5FB, England; e Department of Bioinformatics, School of Chemical and Biotechnology, SASTRA University, Thanjavur 613 401, India

## Abstract

The title salt, C_8_H_12_NO^+^·Br^−^·H_2_O, is isomorphous with the chloride analogue [Seethalakshmi *et al.* (2013). *Acta Cryst*. E**69**, o835–o836]. In the solid state, the cations, anions and water mol­ecules are inter­linked by a network of O—H⋯O, O—H⋯Br and C—H⋯Br inter­actions. The water mol­ecule makes two O—H⋯Br hydrogen bonds, generating [010] zigzag chains of alternating water mol­ecules and bromide anions. The cation is involved in two inter­molecular C—H⋯Cl inter­actions in the chloride salt, whereas three inter­molecular C—H⋯Br inter­actions are observed in the title bromide salt. This additional inter­molecular C—H⋯Br inter­action links the adjacent water and bromide zigzag chains *via* cationic mol­ecules. In addition, weak π–π stacking inter­actions are observed between pyridinium rings [centroid–centroid distance = 3.5664 (13) Å].

## Related literature

For related structures, see: Seethalakshmi *et al.* (2006*a* ▶,*b* ▶,*c* ▶, 2007 ▶, 2013*a* ▶,*b* ▶). For related compounds, see: Dhanuskodi *et al.* (2006 ▶, 2008 ▶). For graph-set motifs, see: Bernstein *et al.* (1995 ▶).
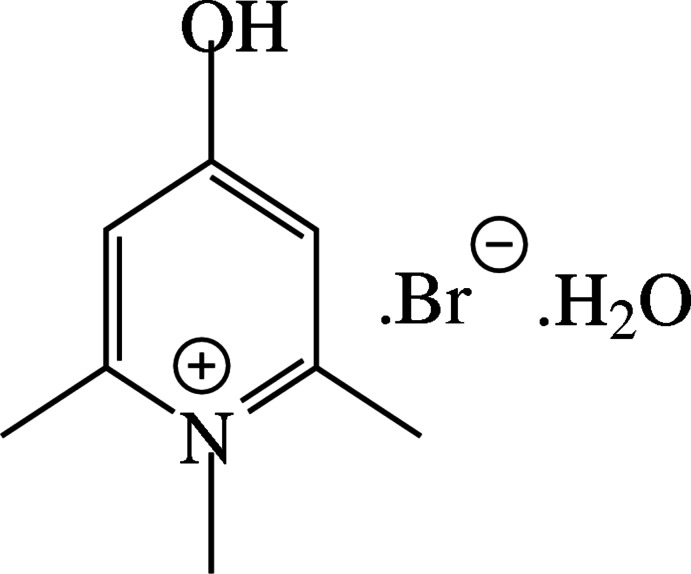


## Experimental

### 

#### Crystal data

C_8_H_12_NO^+^·Br^−^·H_2_O
*M* *_r_* = 236.11
Monoclinic, 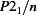

*a* = 8.4796 (4) Å
*b* = 8.5874 (6) Å
*c* = 13.8479 (9) Å
β = 99.504 (4)°
*V* = 994.53 (11) Å^3^
*Z* = 4
Mo *K*α radiation
μ = 4.10 mm^−1^
*T* = 120 K
0.30 × 0.30 × 0.25 mm

#### Data collection

Bruker–Nonius 95mm CCD camera on κ-goniostat diffractometer
Absorption correction: multi-scan (*SADABS*; Sheldrick, 2003 ▶) *T*_min_ = 0.373, *T*_max_ = 0.427
11830 measured reflections
2277 independent reflections
1888 reflections with *I* > 2σ(*I*)
*R*_int_ = 0.037

#### Refinement

*R*[*F* ^2^ > 2σ(*F* ^2^)] = 0.026
*wR*(*F* ^2^) = 0.056
*S* = 1.06
2277 reflections
125 parameters
3 restraints
H atoms treated by a mixture of independent and constrained refinement
Δρ_max_ = 0.59 e Å^−3^
Δρ_min_ = −0.34 e Å^−3^

### 

Data collection: *COLLECT* (Nonius, 1998 ▶); cell refinement: *DENZO* (Otwinowski & Minor, 1997 ▶); data reduction: *DENZO*; method used to solve structure: isomorphous; program(s) used to refine structure: *SHELXL97* (Sheldrick, 2008 ▶); molecular graphics: *PLATON* (Spek, 2009 ▶); software used to prepare material for publication: *SHELXL97*.

## Supplementary Material

Crystal structure: contains datablock(s) I, global. DOI: 10.1107/S1600536813013330/tk5224sup1.cif

Structure factors: contains datablock(s) I. DOI: 10.1107/S1600536813013330/tk5224Isup2.hkl

Supplementary material file. DOI: 10.1107/S1600536813013330/tk5224Isup3.cml

Additional supplementary materials:  crystallographic information; 3D view; checkCIF report

## Figures and Tables

**Table 1:** Hydrogen-bond geometry (Å, °)

*D*—H⋯*A*	*D*—H	H⋯*A*	*D*⋯*A*	*D*—H⋯*A*
O1—H1⋯O1*W* ^i^	0.83 (2)	1.78 (2)	2.607 (2)	174 (3)
O1*W*—H1*W*⋯Br1	0.81 (2)	2.44 (2)	3.2407 (18)	170 (3)
O1*W*—H2*W*⋯Br1^ii^	0.83 (2)	2.43 (2)	3.2527 (18)	168 (3)
C3—H3⋯Br1^i^	0.95	2.86	3.785 (2)	164
C5—H5⋯Br1^iii^	0.95	2.90	3.837 (2)	170
C9—H9*A*⋯Br1^iv^	0.98	2.91	3.822 (2)	155

Symmetry codes: (i) 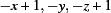; (ii) 
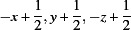
; (iii) 
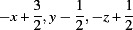
; (iv) 
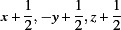
.

**Fig. 1.**
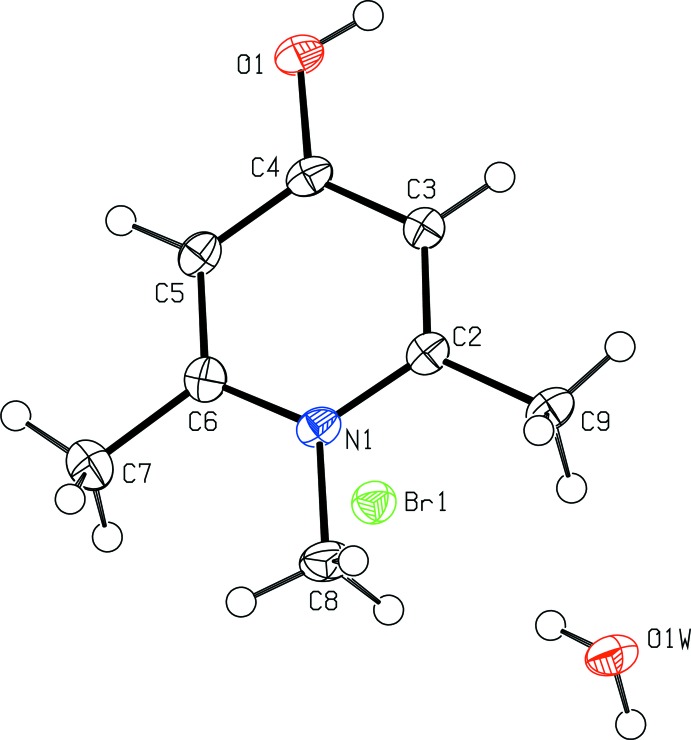
Perspective view of (I), showing the atomic-numbering scheme. The displacement ellipsoids are drawn at the 50% probability level.

**Fig. 2.**
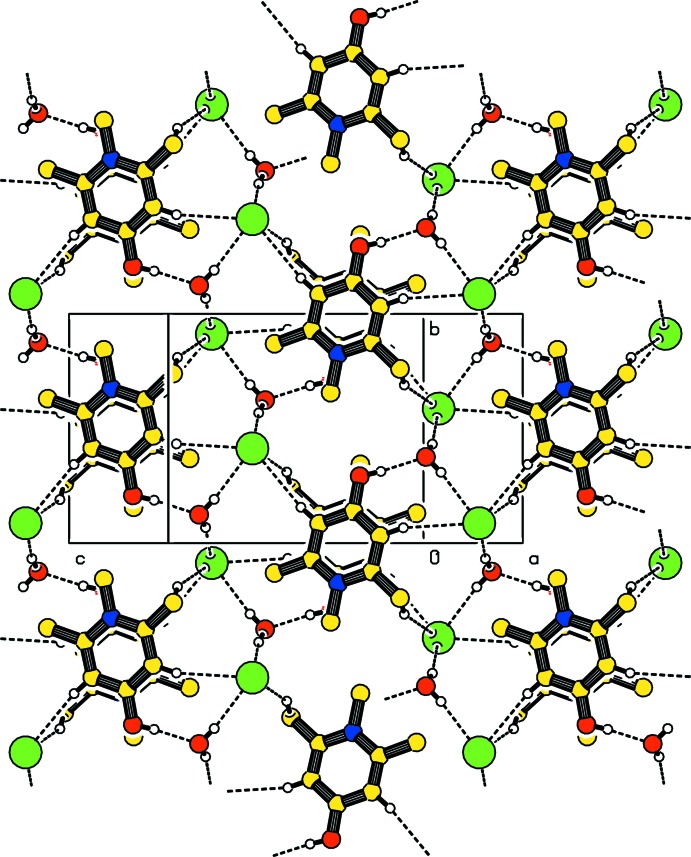
A view of the crystal structure of (I), showing the O—H···O, O—H···Br and C—H···Br interactions indicated as dashed lines.

**Fig. 3.**
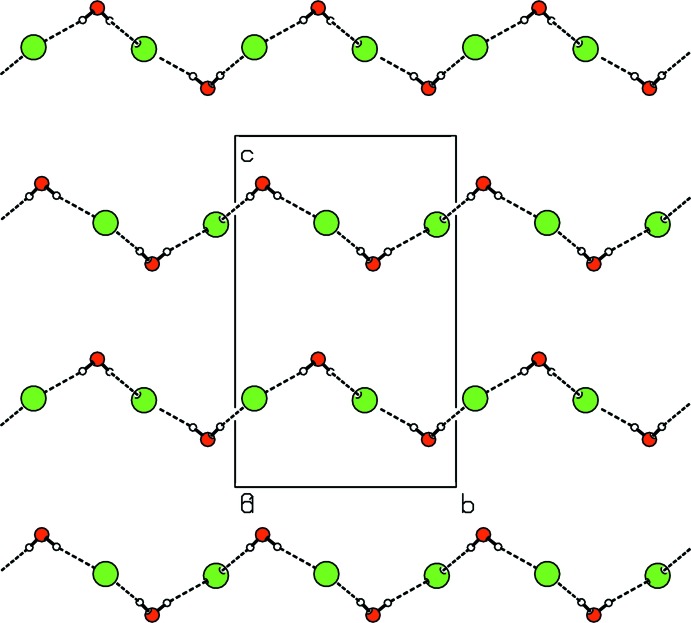
One dimensional zigzag chains generated from alternate water and bromide anion interconnected by O—H···Br hydrogen bond which run parallel to the b axis.

**Fig. 4.**
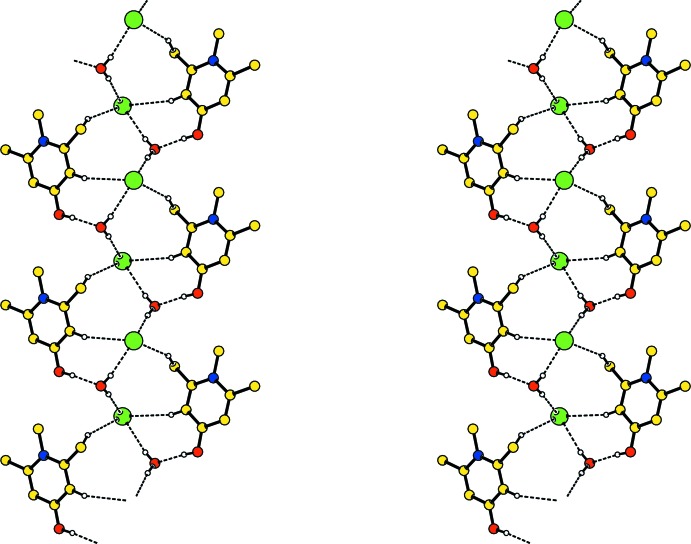
Stereo view of the arrangement of alternate R23(8) and R24(10) ring motifs.

## supplementary crystallographic information

### Comment

In continuation of our studies on pyridinium salts (Seethalakshmi *et al.*, 2006*a*,*b*,*c*; 2007; 2013*a*,*b*; Dhanuskodi *et al.*, 2006; 2008), we determined crystal and molecular structure of 4-hydroxy-1,2,6-trimethylpyridinium bromide monohydrate, (I). This structure is isomorphous with 4-hydroxy-1,2,6-trimethylpyridinium chloride monohydrate (Seethalakshmi *et al.* 2013*a*).

As shown in Fig. 1, the asymmetric unit contains one 4-hydroxy-1,2,6-trimethylpyridinium cation, a bromide anion and a water molecule. The corresponding bond distances and angles of the cation in (I) are comparable with those of related structures (Seethalakshmi *et al.*, 2006*a*,*b*,*c*; 2007; 2013*a*,*b*).

The crystal structure of (I) is stabilized by a network of intermolecular O—H···O, O—H···Br and C—H···Br interactions (Table 1, Fig. 2). In (I), the bromide anions and water molecules are interconnected alternately *via* intermolecular O—H···Br hydrogen bonds. These hydrogen bonds produce a one dimesional zigzag chain which runs parallel to the *b* axis (Fig. 3). The hydroxy group of the cation acts as a donor for an intermolecular O—H···O hydrogen bond with the water molecule. The way two cation molecules are interlinked is the same as observed in the chloride salt (Seethalakshmi *et al.*, 2013*a*). The glide related cations are interconnected by an O—H···O—H···Br···H—O···H—O cooperative hydrogen bonding pattern, whereas cation molecules related by translation are interconnected through another type of O—H···O—H···Br···H—O—H···Br···H—O···H—O cooperative hydrogen bonding mode (Fig. 4).

There are three weak intermolecular C—H···Br (C3—H3···Br, C5—H5···Br and C9—H9A···Br) interactions observed in (I), whereas only two C—H···Cl (C3—H3···Cl and C9—H9A···Cl) interactions are found in the crystal structure of chloride salt (Seethalakshmi *et al.*, 2013*a*). Atom C3 of the cation is involved in a weak C—H···Br intermolecular interaction with bromide anion. As shown in Fig. 4, this weak interaction combines with O—H···O and O—H···Br hydrogen bonds forming a graph-set motif of *R*^2^_3_(8) (Bernstein *et al.*, 1995). One of the methyl atoms C9 (*via* H9A) participates in a weak intermolecular C—H···Br interaction with the bromide anion. Again, this interaction combines with C3—H3···Br and two O—H···Br interactions forming a ring which has a graph-set motif of *R*^2^_4_(10). The *R*^2^_3_(8) and *R*^2^_4_(10) ring motifs are arranged alternately as a helical ribbon which run parallel to the *b* axis (Fig. 4). Atom C5 of the cation (*via* H5) is involved in a weak intermolecular C—H···Br interaction. This additional C—H···Br interaction links the adjacent water and bromide zigzag chains *via* cationic molecules (Fig. 2). In constrast to chloride salt, bromide anion is pentacoordinated by five hydrogen atoms in the crystal structure of (I). The pentacoordination angles in the range of 55–89°. In (I), a weak aromatic π–π stacking interaction is observed between two pyridinium rings related by center of inversion (2 - *x*, -*y*, 1 - *z*) with a centroid-to-centroid distance of 3.5664 (13) Å.

### Experimental

The title salt was prepared by dissolving 1-methyl-2,6-dimethyl-4-hydroxypyridine (1.37 g) with hydrobromic acid (2.43 ml) in distilled water (5 ml). The mixture was stirred at room temperature for 7 h and the clear solution was kept for evaporation at 60 °C after filtration. Finally crystalline powder was obtained and dissolved in double distilled water. Single crystals suitable for X-ray diffraction were obtained by slow evaporation.

### Refinement

Since the title salt is isomorphous with its chloride counterpart, it was refined with the coordinates of the cation moiety of chloride salt (Seethalakshmi *et al.*, 2013*a*). The positions of the Br atom and water molecule were determined from a difference Fourier map and refined anisotropically. The positions of hydroxy H atom and H atoms of water molecule were determined from a difference Fourier map and refined freely along with their isotropic displacement parameters. In the final round of refinement, the O—H bond lengths of the water molecule and hydroxy group are restrained to 0.84 (2) Å. The methyl H atoms were constrained to an ideal geometry (C—H = 0.98 Å), with *U*_iso_(H) = 1.5*U*_eq_(C), but were allowed to rotate freely about the C—C and N—C bonds. The remaining H atoms were placed in geometrically idealized positions (C—H = 0.95 Å), with *U*_iso_(H) = 1.2*U*_eq_(C) and were constrained to ride on their parent atoms.

### Crystal data

**Table:**

C_8_H_12_NO^+^·Br^−^·H_2_O	*F*(000) = 480
*M**_r_* = 236.11	*D*_x_ = 1.577 Mg m^−^^3^
Monoclinic, *P*2_1_/*n*	Mo *K*α radiation, λ = 0.71073 Å
Hall symbol: -P 2yn	Cell parameters from 2311 reflections
*a* = 8.4796 (4) Å	θ = 1–27.5°
*b* = 8.5874 (6) Å	µ = 4.10 mm^−^^1^
*c* = 13.8479 (9) Å	*T* = 120 K
β = 99.504 (4)°	Block, colourless
*V* = 994.53 (11) Å^3^	0.30 × 0.30 × 0.25 mm
*Z* = 4	

### Data collection

**Table:**

Bruker–Nonius 95mm CCD camera on κ-goniostat diffractometer	2277 independent reflections
Radiation source: Bruker–Nonius FR591 rotating anode	1888 reflections with *I* > 2σ(*I*)
Graphite monochromator	*R*_int_ = 0.037
Detector resolution: 9.091 pixels mm^-1^	θ_max_ = 27.5°, θ_min_ = 2.8°
φ and ω scans	*h* = −9→10
Absorption correction: multi-scan (*SADABS*; Sheldrick, 2003)	*k* = −11→11
*T*_min_ = 0.373, *T*_max_ = 0.427	*l* = −17→17
11830 measured reflections	

### Refinement

**Table:**

Refinement on *F*^2^	Secondary atom site location: difference Fourier map
Least-squares matrix: full	Hydrogen site location: inferred from neighbouring sites
*R*[*F*^2^ > 2σ(*F*^2^)] = 0.026	H atoms treated by a mixture of independent and constrained refinement
*wR*(*F*^2^) = 0.056	*w* = 1/[σ^2^(*F*_o_^2^) + (0.0179*P*)^2^ + 0.9223*P*] where *P* = (*F*_o_^2^ + 2*F*_c_^2^)/3
*S* = 1.06	(Δ/σ)_max_ = 0.001
2277 reflections	Δρ_max_ = 0.59 e Å^−^^3^
125 parameters	Δρ_min_ = −0.34 e Å^−^^3^
3 restraints	Extinction correction: *SHELXL97* (Sheldrick, 2008), Fc^*^=kFc[1+0.001xFc^2^λ^3^/sin(2θ)]^-1/4^
Primary atom site location: structure-invariant direct methods	Extinction coefficient: 0.0025 (5)

### Special details

**Table:**

Experimental. The minimum and maximum absorption values stated above are those calculated in *SHELXL97* from the given crystal dimensions. The ratio of minimum to maximum apparent transmission was determined experimentally as 0.696421.
Geometry. All e.s.d.'s (except the e.s.d. in the dihedral angle between two l.s. planes) are estimated using the full covariance matrix. The cell e.s.d.'s are taken into account individually in the estimation of e.s.d.'s in distances, angles and torsion angles; correlations between e.s.d.'s in cell parameters are only used when they are defined by crystal symmetry. An approximate (isotropic) treatment of cell e.s.d.'s is used for estimating e.s.d.'s involving l.s. planes.
Refinement. Refinement of *F*^2^ against ALL reflections. The weighted *R*-factor *wR* and goodness of fit *S* are based on *F*^2^, conventional *R*-factors *R* are based on *F*, with *F* set to zero for negative *F*^2^. The threshold expression of *F*^2^ > σ(*F*^2^) is used only for calculating *R*-factors(gt) *etc*. and is not relevant to the choice of reflections for refinement. *R*-factors based on *F*^2^ are statistically about twice as large as those based on *F*, and *R*- factors based on ALL data will be even larger.

### Fractional atomic coordinates and isotropic or equivalent isotropic displacement parameters (Å^2^)

**Table:**

	*x*	*y*	*z*	*U*_iso_*/*U*_eq_	
Br1	0.46272 (3)	0.08691 (3)	0.252474 (16)	0.02160 (9)	
O1	0.8002 (2)	−0.29194 (18)	0.48421 (13)	0.0265 (4)	
O1W	0.3258 (2)	0.3759 (2)	0.36438 (13)	0.0270 (4)	
N1	0.7983 (2)	0.1745 (2)	0.42283 (14)	0.0185 (4)	
C2	0.7227 (2)	0.1241 (2)	0.49673 (16)	0.0175 (4)	
C3	0.7211 (3)	−0.0319 (3)	0.51871 (16)	0.0185 (5)	
H3	0.6689	−0.0670	0.5703	0.022*	
C4	0.7960 (3)	−0.1390 (3)	0.46562 (17)	0.0196 (5)	
C5	0.8699 (3)	−0.0850 (3)	0.38918 (16)	0.0199 (5)	
H5	0.9208	−0.1565	0.3519	0.024*	
C6	0.8691 (2)	0.0712 (3)	0.36768 (16)	0.0190 (5)	
C7	0.9436 (3)	0.1302 (3)	0.28400 (18)	0.0278 (5)	
H7A	0.8608	0.1764	0.2346	0.042*	
H7B	0.9945	0.0436	0.2548	0.042*	
H7C	1.0241	0.2091	0.3078	0.042*	
C8	0.7984 (3)	0.3436 (3)	0.4015 (2)	0.0284 (6)	
H8A	0.6880	0.3802	0.3832	0.043*	
H8B	0.8572	0.3627	0.3474	0.043*	
H8C	0.8501	0.3999	0.4598	0.043*	
C9	0.6442 (3)	0.2398 (3)	0.55408 (17)	0.0236 (5)	
H9A	0.7252	0.3102	0.5889	0.035*	
H9B	0.5904	0.1849	0.6015	0.035*	
H9C	0.5654	0.3002	0.5094	0.035*	
H1	0.756 (3)	−0.313 (3)	0.5317 (17)	0.038 (9)*	
H1W	0.355 (3)	0.309 (3)	0.330 (2)	0.044 (9)*	
H2W	0.261 (4)	0.432 (3)	0.328 (2)	0.062 (11)*	

### Atomic displacement parameters (Å^2^)

**Table:**

	*U*^11^	*U*^22^	*U*^33^	*U*^12^	*U*^13^	*U*^23^
Br1	0.02104 (13)	0.01973 (12)	0.02404 (14)	−0.00007 (9)	0.00380 (8)	0.00019 (10)
O1	0.0352 (10)	0.0132 (8)	0.0329 (10)	0.0028 (7)	0.0108 (8)	0.0001 (7)
O1W	0.0316 (10)	0.0181 (9)	0.0307 (10)	0.0045 (7)	0.0037 (8)	0.0019 (8)
N1	0.0166 (9)	0.0138 (9)	0.0242 (11)	−0.0018 (7)	0.0010 (7)	−0.0019 (7)
C2	0.0132 (10)	0.0179 (11)	0.0201 (12)	−0.0013 (8)	−0.0015 (8)	−0.0028 (9)
C3	0.0174 (11)	0.0195 (11)	0.0181 (12)	−0.0001 (8)	0.0014 (9)	−0.0010 (9)
C4	0.0177 (11)	0.0157 (10)	0.0240 (12)	0.0007 (8)	−0.0007 (9)	−0.0013 (9)
C5	0.0177 (11)	0.0185 (10)	0.0229 (12)	0.0004 (9)	0.0013 (9)	−0.0044 (9)
C6	0.0143 (10)	0.0216 (11)	0.0203 (11)	−0.0026 (9)	0.0002 (8)	−0.0021 (9)
C7	0.0273 (13)	0.0305 (13)	0.0268 (13)	−0.0025 (10)	0.0079 (10)	0.0022 (10)
C8	0.0300 (13)	0.0153 (11)	0.0407 (16)	0.0003 (10)	0.0081 (11)	0.0041 (10)
C9	0.0254 (12)	0.0179 (11)	0.0268 (13)	0.0035 (9)	0.0024 (9)	−0.0033 (10)

### Geometric parameters (Å, º)

**Table:**

O1—C4	1.338 (3)	C5—C6	1.374 (3)
O1—H1	0.828 (17)	C5—H5	0.9500
O1W—H1W	0.813 (17)	C6—C7	1.496 (3)
O1W—H2W	0.833 (18)	C7—H7A	0.9800
N1—C2	1.365 (3)	C7—H7B	0.9800
N1—C6	1.372 (3)	C7—H7C	0.9800
N1—C8	1.482 (3)	C8—H8A	0.9800
C2—C3	1.374 (3)	C8—H8B	0.9800
C2—C9	1.496 (3)	C8—H8C	0.9800
C3—C4	1.394 (3)	C9—H9A	0.9800
C3—H3	0.9500	C9—H9B	0.9800
C4—C5	1.395 (3)	C9—H9C	0.9800
			
C4—O1—H1	111 (2)	C5—C6—C7	120.7 (2)
H1W—O1W—H2W	107 (3)	C6—C7—H7A	109.5
C2—N1—C6	121.01 (18)	C6—C7—H7B	109.5
C2—N1—C8	118.45 (19)	H7A—C7—H7B	109.5
C6—N1—C8	120.52 (19)	C6—C7—H7C	109.5
N1—C2—C3	119.9 (2)	H7A—C7—H7C	109.5
N1—C2—C9	119.51 (19)	H7B—C7—H7C	109.5
C3—C2—C9	120.6 (2)	N1—C8—H8A	109.5
C2—C3—C4	120.3 (2)	N1—C8—H8B	109.5
C2—C3—H3	119.9	H8A—C8—H8B	109.5
C4—C3—H3	119.9	N1—C8—H8C	109.5
O1—C4—C3	123.1 (2)	H8A—C8—H8C	109.5
O1—C4—C5	118.1 (2)	H8B—C8—H8C	109.5
C3—C4—C5	118.8 (2)	C2—C9—H9A	109.5
C6—C5—C4	120.1 (2)	C2—C9—H9B	109.5
C6—C5—H5	119.9	H9A—C9—H9B	109.5
C4—C5—H5	119.9	C2—C9—H9C	109.5
N1—C6—C5	119.8 (2)	H9A—C9—H9C	109.5
N1—C6—C7	119.5 (2)	H9B—C9—H9C	109.5
			
C6—N1—C2—C3	2.1 (3)	O1—C4—C5—C6	−179.7 (2)
C8—N1—C2—C3	−179.6 (2)	C3—C4—C5—C6	0.4 (3)
C6—N1—C2—C9	−178.86 (19)	C2—N1—C6—C5	−2.6 (3)
C8—N1—C2—C9	−0.6 (3)	C8—N1—C6—C5	179.2 (2)
N1—C2—C3—C4	−0.3 (3)	C2—N1—C6—C7	176.9 (2)
C9—C2—C3—C4	−179.33 (19)	C8—N1—C6—C7	−1.4 (3)
C2—C3—C4—O1	179.2 (2)	C4—C5—C6—N1	1.3 (3)
C2—C3—C4—C5	−1.0 (3)	C4—C5—C6—C7	−178.1 (2)

### Hydrogen-bond geometry (Å, º)

**Table:**

*D*—H···*A*	*D*—H	H···*A*	*D*···*A*	*D*—H···*A*
O1—H1···O1*W*^i^	0.83 (2)	1.78 (2)	2.607 (2)	174 (3)
O1*W*—H1*W*···Br1	0.81 (2)	2.44 (2)	3.2407 (18)	170 (3)
O1*W*—H2*W*···Br1^ii^	0.83 (2)	2.43 (2)	3.2527 (18)	168 (3)
C3—H3···Br1^i^	0.95	2.86	3.785 (2)	164
C5—H5···Br1^iii^	0.95	2.90	3.837 (2)	170
C9—H9*A*···Br1^iv^	0.98	2.91	3.822 (2)	155

Symmetry codes: (i) −*x*+1, −*y*, −*z*+1; (ii) −*x*+1/2, *y*+1/2, −*z*+1/2; (iii) −*x*+3/2, *y*−1/2, −*z*+1/2; (iv) *x*+1/2, −*y*+1/2, *z*+1/2.
